# Saliva viral load better correlates with clinical and immunological profiles in children with coronavirus disease 2019

**DOI:** 10.1080/22221751.2021.1878937

**Published:** 2021-02-20

**Authors:** Gilbert T. Chua, Joshua S. C. Wong, Kelvin K. W. To, Ivan C. S. Lam, Felix Y. S. Yau, Wai Hung Chan, Polly P. K. Ho, Jaime S. R. Duque, Cyril C. Y. Yip, Anthony C. K. Ng, Wilfred H. S. Wong, Joyce H. Y. Kwong, Kate F. S. Leung, P. T. Wan, Kelly Lam, Ian C. K. Wong, Janette Kwok, Marco H. K. Ho, Godfrey C. F. Chan, Yu Lung Lau, Patrick Ip, Mike Y. W. Kwan

**Affiliations:** a Department of Paediatrics and Adolescent Medicine, Li Ka Shing Faculty of Medicine, The University of Hong Kong, Hong Kong SAR, People’s Republic of China; b Paediatric Infectious Disease Unit, Department of Paediatrics and Adolescent Medicine, Princess Margaret Hospital, Hong Kong SAR, People’s Republic of China; c Department of Microbiology, Carol Yu Centre for Infection, Li Ka Shing Faculty of Medicine, The University of Hong Kong, Hong Kong SAR, People’s Republic of China; d Department of Paediatrics, Queen Elizabeth Hospital, Hong Kong SAR, People’s Republic of China; e Haematology laboratory, Department of Pathology, Princess Margaret Hospital, Hong Kong SAR, People’s Republic of China; f Centre for Safe Medication Practice and Research, Department of Pharmacology and Pharmacy, The University of Hong Kong, Hong Kong SAR, People’s Republic of China; g Research Department of Practice and Policy, UCL School of Pharmacy, University College, London, United Kingdom; h Divison of Transplantation and Immunogenetics, Department of Pathology, Queen Mary Hospital, HKSAR, People’s Republic of China

**Keywords:** COVID-19, children, saliva, immunological profiles, viral load

## Abstract

**Background:**

Pediatric COVID-19 studies exploring the relationships between NPS and saliva viral loads, clinical and immunological profiles are lacking.

**Methods:**

Demographics, immunological profiles, nasopharyngeal swab (NPS), and saliva samples collected on admission, and hospital length of stay (LOS) were assessed in children below 18 years with COVID-19.

**Findings:**

91 patients were included between March and August 20 20. NPS and saliva viral loads were correlated (*r* = 0.315, *p* = 0.01). Symptomatic patients had significantly higher NPS and saliva viral loads than asymptomatic patients. Serial NPS and saliva viral load measurements showed that the log_10_ NPS (*r* = −0.532, *p* < 0.001) and saliva (*r* = −0.417, *p* < 0.001) viral loads for all patients were inversely correlated with the days from symptom onset with statistical significance. Patients with cough, sputum, and headache had significantly higher saliva, but not NPS, viral loads. Higher saliva, but not NPS, viral loads were associated with total lymphopenia, CD3 and CD4 lymphopenia (all *p* < 0.05), and were inversely correlated with total lymphocyte (*r* = −0.43), CD3 (*r* = −0.55), CD4 (*r* = −0.60), CD8 (*r* = −0.41), B (*r* = −0.482), and NK (*r* = −0.416) lymphocyte counts (all *p* < 0.05).

**Interpretation:**

Saliva viral loads on admission in children correlated better with clinical and immunological profiles than NPS.

## Introduction

Since the coronavirus disease 2019 (COVID-19) outbreak in late December 2019, more than 81 million people have been infected globally and more than 1.8 million people have died [1]. Studies in Asia reported the majority of children with COVID-19 had only mild symptoms [2,3]. Children with COVID-19 had various symptoms including fever, pneumonia, and low lymphocyte count that were associated with prolonged viral shedding [4], whereas their immunoglobulin G (IgG) and neutrophil to lymphocyte ratio were negatively associated with liver and myocardial enzymes [5]. However, clinical studies focusing on the association between clinical outcomes and immunological profiles in children with COVID-19 are lacking. Furthermore, the association between respiratory tract viral load and immunological profiles has yet to be explored.

Immunological studies showed that adults with COVID-19 with lymphopenia and suppressed T lymphocyte subsets and NK cells were predicted to have a more severe infection [6–8]. Furthermore, severe patients had a higher ratio of conventional dendritic cells to plasmacytoid cells [9]. A recent adult study demonstrated that saliva viral load was reliable for the diagnosis and monitoring of SARS-CoV-2 [10,11], and that saliva was possibly superior to nasopharyngeal swabs (NPS) samples with less variation and false-negative results [12]. However, many studies exploring the viral load burden in children have only used NPS samples [13]. SARS-CoV-2 RNA was detectable during the early phase of infection in mildly symptomatic and symptomatic children [14], and saliva viral load was also the highest around the time of presentation [15]. Therefore, comparing the clinical utility and correlation of viral loads in NPS and saliva in children with COVID-19 is an important research question that needs to be answered. This study aimed to determine the relationships between the clinical symptoms, immunological profiles, viral loads in NPS and saliva, and hospital length of stay (LOS) in children with COVID-19, as well as compare symptomatic and asymptomatic patients.

## Methods

### Patient recruitment and clinical data collection

Pediatric patients below 18 years of age who tested positive for SARS-CoV-2 by reverse transcriptase polymerase chain reaction (RT–PCR) of respiratory specimens and admitted to the Princess Margaret Hospital or Queen Elizabeth Hospital in Hong Kong between 12 March and 8 August 2020 were included in the analysis. Asymptomatic patients, detected by screening at the border or by contract tracing, were also admitted as part of the territory-wide infection control policy in Hong Kong. Demographic details including age, gender, symptoms, travel history, and length of hospital stay were collected. Laboratory tests performed on admission included complete blood count (CBC), renal and liver function tests, immunoglobulin G/A/M (IgG/IgA/IgM) levels, and lymphocyte subsets. NPS was obtained for the detection of the presence of SARS-CoV-2 RNA. NPS and saliva were also collected for viral load measurements on admission and were repeated every five to seven days until discharge. Saliva for viral load quantification was collected for children ≥ 3 years old by spitting saliva into a sterile bottle in the morning before brushing teeth and having breakfast. Only NPS will be collected for viral load study if they were unable to provide saliva specimens, especially for younger children.

### Clinical definitions

Lymphopenia or lymphocytosis, including subsets, were defined as lower than the 10th and higher than the 90^th^ percentile based on the Chinese pediatric age and gender-matched reference ranges [16]. Clinical and laboratory profiles were compared between patients with lymphopenia and lymphocytosis. For discharge criteria before 6 July 2020, patients with COVID-19 should have two consecutive negative RT–PCR results for SARS-CoV-2 taken at least 24 h apart before they could be discharged from hospital. Patients testing positive had another repeat RT–PCR test every 2–3 days until the NPS results were negative. For discharge criteria after 6 July 2020, all patients with COVID-19 were allowed to be discharged from isolation facilities with detectable SARS-CoV-2 anti-nucleoprotein (NP) IgG antibodies in their serum regardless of a positive RT–PCR result of a respiratory specimen [17]. Anti-NP IgG measurements were performed on day 5 or day 10 after admission for asymptomatic patients and symptomatic patients, respectively. If no antibodies were detected, serology was repeated every 2–3 days.

### Laboratory tests

Nasopharyngeal swabs obtained during admission and for monitoring of the presence of SARS-CoV-2 were tested by RT–PCR using a LightMix® Modular SARS and Wuhan CoV E-gene kit (TIB Molbiol, Berlin, Germany) on a LightCycler Multiplex RNA Virus Master (Roche, Penzberg, Germany) according to the manufacturer’s instructions as previously described [18]. SARS-CoV-2 viral load analysis of NPS and saliva samples were determined by RT–PCR assay developed by the University of Hong Kong targeting the RNA-dependent RNA polymerase /helicase gene of SARS-CoV-2 (*COVID-19-RdRp/Hel*). Details of the test have been published elsewhere [19]. The lymphocyte subsets were determined by immunophenotyping of peripheral blood lymphocytes using a single platform with a lyse-no-wash procedure on a FC500 flow cytometer (Beckman Coulter, Brea, California, USA). The lymphocyte subsets included total T cells (CD3+), helper T cells (CD3+CD4+), cytotoxic T cells (CD3+CD8+), total B cells (CD19+), and total NK cells (CD3-CD16/CD56+). SARS-CoV-2 anti-NP IgG was measured using a chemiluminescent microparticle immunoassay for the qualitative detection of IgG in patient serum against the SARS-CoV-2 nucleoprotein (Abbott, Abbott Park, Illinois, USA) [20].

### Ethics approval

This study was approved by the University of Hong Kong/Hospital Authority Hong Kong West Cluster Institutional Review Board (reference number: UW 20-292), the Kowloon West Cluster Research Ethics Committee (reference number: KW/FR-20-086) and the Kowloon Central Cluster Research Ethics Committee (reference number: KC/KE-20-0140/ER-1). Individual informed consent was not required as it is a retrospective study and all patients’ data were kept anonymous.

### Statistical analysis

Statistical analyses were performed using SPSS software version 19 (Armonk, New York, USA), Microsoft Excel (Microsoft, Redmond, Washington, USA) and Statistical Analysis System v9.4 (SAS Institute Inc., Cary, NC). Continuous variables were expressed as mean and interquartile ranges (IQR) and analyzed by independent t-test or Mann–Whitney U test where appropriate. Categorical variables were expressed as number (%) and analyzed by Chi-square test or Fisher exact test. Unpaired T-test was performed to analyze the association between the clinical symptoms and the log_10_ of viral load. Pearson correlation was performed to analyze the relationship between the laboratory results and the log_10_ viral load. A two-tailed *p*-value less than 0.05 was considered statistically significant.

## Results

### Demographics and clinical profiles

Between 12 March and 8 August 2020, 91 children with COVID-19 were admitted to the Princess Margaret Hospital and the Queen Elizabeth Hospital in Hong Kong. The mean age was 9.01 years (IQR 4 - 13 years, range 0.1 - 18 years); 48.4% (44/91) were male; 30.8% (28/91) were asymptomatic. The majority had no significant past medical history. One patient had asthma, one had attention deficit hyperactivity disorder, one had repaired coarctation of the aorta, one had epilepsy, and one had tuberous sclerosis. Most commonly reported symptoms included fever (38.5%; 35/91), cough (23.1%), rhinorrhea (19.8%), and sputum production (12.1%). Five patients reported ageusia and four patients reported anosmia, of which three patients reported suffering from both symptoms. None required intensive care or mechanical ventilation, and no mortality was reported. Among all patients, 28 (30.8%) symptomatic patients received combination antiviral therapy lopinavir/ritonavir (Kaletra®), and nine asymptomatic patients also received empirical treatment. At the time of the analysis, 27 and 56 patients were discharged before and after 6 July 2020, respectively, and eight were still in hospital. Symptomatic patients discharged before 6 July 2020 had significantly longer LOS (27.1 vs. 16.3 days, *p* = 0.024). There were no significant differences between symptomatic and asymptomatic patients discharged after 6 July 2020 in terms of LOS (Table 1).

**Table 1.** Comparison between asymptomatic and symptomatic children with COVID-19.

	Asymptomatic****Mean (IQR)*****N* = 28	Symptomatic****Mean (IQR)*****N* = 63	*p*-values
Age (years)	8.6 (4.3–11.0)	9.2 (4.0–15.0)	0.658
Male	16 (57.1%)	28 (44.4%)	0.364
Treatment (lopinavir/ritonavir)	9 (32.1%)	19 (30.6%)	1.000
Length of Stay (days)			
Discharged on or before 6 July 2020	16.3 (10.5–21.5)	27.1 (16.5–38.5)	**0**.024*
Discharged after 6 July 2020	12.4 (11.0–14.0)	11.4 (9.0–14.3)	0.296
NPS (log_10_ copies/mL)	5.7 (4.3–7.4)	6.8 (5.6–8.3)	**0**.014*
Saliva (log_10_ copies/mL)	4.5 (3.6–5.1)	5.8 (4.9–6.7)	**0**.002*
Total white cell count (x 10^9^/L)	6.0 (7.4–4.1)	5.8 (4.2–6.4)	0.751
Hemoglobin (g/dL)	12.8 (13.5–12.0)	13.2 (14.3–12.4)	0.250
Platelets (x 10^9^/L)	258.4 (298.0–210.0)	278.1 (317.0–215.0)	0.331
Neutrophil (x 10^9^/L)	2.5 (1.5–2.9)	2.5 (1.7–3.2)	0.950
Lymphocytes (x 10^9^/L)	2.9 (1.6–3.8)	2.7 (1.6–2.8)	0.558
Monocyte (x 10^9^/L)	0.5 (0.3–0.6)	0.5 (0.4–0.6)	0.645
CD3+ T cells (cells/µL)	2049.5 (1165.—2367.0)	1557.1 (1012.0–1690.5)	0.235
CD4+ Helper T cells (cells/µL)	1020.5 (628.0–1238.0)	775.7 (439.8–849.8)	0.221
CD8+ Cytotoxic T Cells (cells/µL)	792.8 (482.0–874.0)	543.5 (358.8–592.0)	0.132
B Cells (cells/µL)	433.1 (209.0–653.0)	359.3 (185.8–372.3)	0.584
Natural Killer Cells (cells/uL)	330.1 (117.0–444.0)	288.6 (109.5–404.8)	0.647
Urea (mmol/L)	3.4 (2.6–4.2)	3.9 (3.0–4.7)	0.081
Creatinine (µmol/L)	41.6 (32.0–49.0)	44.9 (29.0–57.0)	0.383
Creatine Kinase (U/L)	122.5 (69.0–113.0)	99.7 (62.0–117.5)	0.477
Troponin I (ng/L)	1.9 (1.9–1.9)	11.3 (1.9–10.5)	0.198
Lactate Dehydrogenase (U/L)	219.5 (181.0–249.0)	220.0 (179.0–265.0)	0.967
Alanine Aminotransferase (U/L)	18.0 (13.0–22.0)	20.1 (12.0–21.0)	0.543
Alkaline Phosphatase (U/L)	180.7 (113.8–227.8)	194.8 (92.0–237.0)	0.668
Albumin (g/L)	38.9 (37.0–41.0)	40.2 (38.0–42.0)	0.064
Globulin (g/L)	33.0 (30.0–35.0)	34.0 (32.0–36.3)	0.268
Immunoglobulin G (g/L)	14.9 (9.0–13.5)	11.0 (9.5–13.3)	0.179
Immunoglobulin A (g/L)	1.4 (0.8–1.8)	1.5 (0.8–1.9)	0.571
Immunoglobulin M (g/L)	1.2 (0.7–1.5)	1.3 (0.9–1.7)	0.591
Erythrocyte Sedimentation Rate (mm/hr)	8.6 (2.5–13.0)	12.0 (5.0–15.3)	0.370
C Reactive Protein (mg/dL)	1.4 (0.7–1.0)	1.7 (0.7–2.0)	0.495

• *p* < 0.05.

### Immunological profiles

Based on age- and gender-matched lymphocyte and lymphocyte subset references, 41 patients had total lymphopenia, 20 had CD3 lymphopenia, 15 had CD4 lymphopenia, 24 had CD8 lymphopenia, 19 had B lymphopenia, and 21 had NK lymphopenia. Children with total lymphopenia were significantly older (mean age 10.4 vs. 7.9 years, *p* = 0.027). Only a very small number of children had lymphocytosis. There were no significant differences between the lymphocytosis and non-lymphocytosis groups.

### NPS and saliva viral load profiles

**Figure 1.**
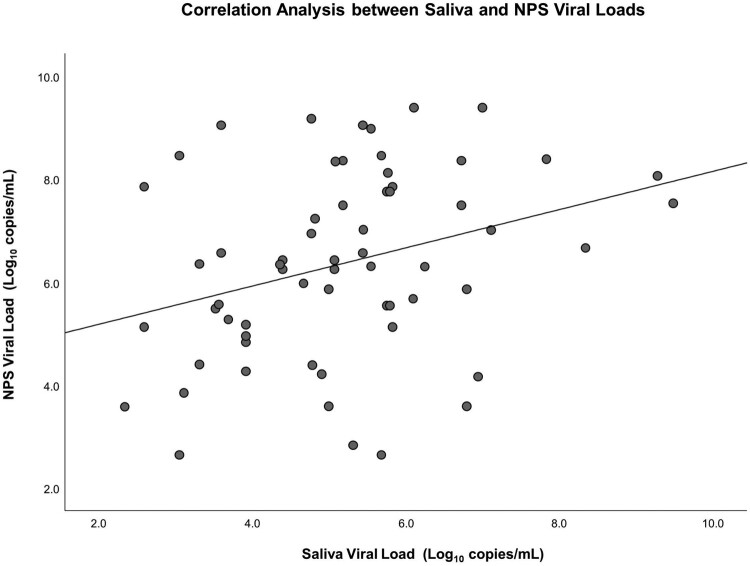
Correlation analysis between saliva and nasopharyngeal swab (NPS) viral loads. Saliva and NPS viral load were correlated with statistical significance. (*r* = 0.315, *p* = 0.01).

On admission, 42 patients had both NPS and saliva viral loads measured. The concordance of NPS and saliva viral load was 100%. There was a significant correlation (*r* = 0.315, *p* = 0.01) between NPS and saliva log_10_ viral loads (Figure 1). Symptomatic patients had significantly higher NPS viral loads (mean log_10_ viral load 6.8 vs. 5.7 copies/mL, *p* = 0.014) and higher saliva viral loads (mean log_10_ viral load 5.8 vs. 4.5 copies/mL, *p* = 0.002) compared to those of asymptomatic patients. (Table 1) Patients with cough (mean log_10_ viral load 6.4 vs. 4.9 copies/mL, *p* = 0.002), sputum production (mean log_10_ viral load 6.4 vs. 5.2 copies/mL, *p* = 0.036), and headache (mean log_10_ viral load 7.3 vs. 5.2 copies/mL, *p* = 0.008) had higher saliva viral loads than those without the symptoms (Supplementary file).

### Correlation between serial viral load measurements and days from symptom onset

**Figure 2.**
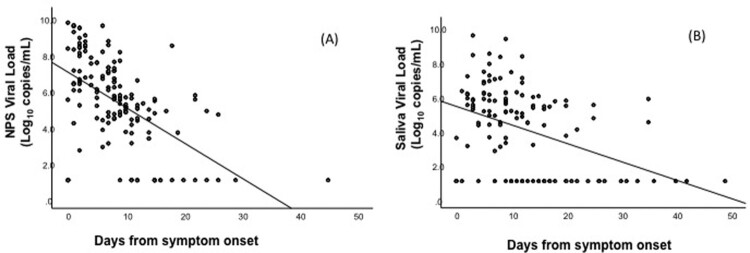
Correlation between serial nasopharyngeal swab (NPS) and saliva viral loads, and the days from symptom onset. The log_10_ viral loads for all patients were negatively correlated with the days from symptom onset. (A) NPS (*r* = −0.532, *p* < 0.001) (B) Saliva (*r* = −0.417, *p* < 0.001).

Figure 2 showed the serial NPS and saliva viral loads measurements of all patients against the symptom onset date presented in scatterplot manner. The log_10_ NPS (*r* = −0.532, *p* < 0.001) and Saliva (*r* = −0.417, *p* < 0.001) viral loads for all patients were inversely correlated with the days from symptom onset with statistical significance.

### Correlation between immunological profiles and viral loads

Significantly higher saliva viral load was observed in children with total lymphopenia (mean log_10_ viral load 5.8 vs. 4.9 copies/mL, *p* = 0.031), CD3 lymphopenia (mean log_10_ viral load 6.1 vs. 5.1 copies/mL, *p* = 0.031), and CD4 lymphopenia (mean log_10_ viral load 6.4 vs. 5.1 copies/mL, *p* = 0.013) compared to those without lymphopenia (Table 2). Saliva viral load on admission was inversely correlated with total lymphocyte (*r* = −0.43, *p* = 0.001), CD3 cell counts (*r* = −0.55, *p* = 0.005), CD4 cell counts (*r* = −0.60, *p* = 0.002), CD8 (*r* = −0.41, *p* = 0.049), B cell counts (*r* = −0.482, *p* = 0.017) and NK cell counts (*r* = −0.416, *p* = 0.043). There were no significant correlations in NPS viral loads across the immunological profiles or with any blood results (Supplementary File). We performed subgroup analysis for the 42 patients who had both NPS and saliva viral load measured. The correlation remained significant between saliva viral load and total lymphocyte (*r* = −0.381, *p* = 0.013), CD3 cell counts (*r* = −0.702, *p* = 0.003), CD4 cell counts (*r* = −0.692, *p* = 0.004), CD8 cell counts (*r* = −0.710, *p* = 0.003) and B cell counts (*r* = −0.515, *p* = 0.049), but not between NPS viral load and their lymphocyte subsets.

**Table 2.** Comparison of nasopharyngeal swab (NPS) and saliva viral loads for patients with lymphopenia and non-lymphopenia.

** **	** **	Lymphopenia		Non-lymphopenia		Sig. (2-tailed)
		N	Mean (IQR)	N	Mean (IQR)	
**Viral load (Log_10_/ copies/mL)**	NPS	36	6.7 (5.5–8.3)	42	6.2 (4.3–7.8)	0.187
	Saliva	26	5.8 (4.7–7.0)	28	4.9 (3.9–5.8)	**0**.031*
** **		T cell lymphopenia		Non-T cell lymphopenia		Sig. (2-tailed)
		N	Mean (IQR)	N	Mean (IQR)	
**Viral load (Log_10_/ copies/mL)**	NPS	15	7.1 (6.0–8.3)	63	6.3 (4.7–7.9)	0.165
	Saliva	14	6.1 (5.0–7.3)	40	5.1 (3.9–5.8)	**0**.031*
** **	** **	CD4 Lymphopenia		Non-CD4 lymphopenia		Sig. (2-tailed)
		N	Mean (IQR)	N	Mean (IQR)	
**Viral load (Log_10_/ copies/mL)**	NPS	11	7.3 (6.7–8.4)	67	6.4 (4.8–7.9)	0.114
	Saliva	10	6.4 (5.0–7.8)	44	5.1 (3.9–5.8)	**0**.013*

Lymphopenia refers to < 10th percentile of age and gender-matched reference. *p < 0.05.

## Discussion

To date, this is one of the largest pediatric COVID-19 studies to explore the relationship between the clinical outcomes, immunological profiles, and NPS and saliva viral loads. Our study demonstrated that saliva viral load, but not NPS viral load, had better correlations with clinical symptoms, hospital LOS and immunological profiles.

Children with respiratory symptoms and headache were more likely to have higher saliva viral loads, but there were no significant differences in their NPS viral loads. Pediatric cross-sectional studies have shown that approximately 30% – 50% children did not present with fever [2,21], which suggested NPS viral load might not be the best to correlate with the clinical symptoms and immunological profiles. In a recent study by Chong et al with a smaller sample size of 18 children that compared cycle threshold values of SARS-CoV-2 in NPS and saliva, they concluded that saliva was not useful for the diagnosis of children with COVID-19 [22]. Conversely, saliva was also shown to be a reliable specimen for the detection of SARS-CoV-2 by RT–PCR [12], and using saliva for the detection of SARS-CoV-2 RNA had less variability and less false negatives [23]. Yonker et al. reported that children with COVID-19 had significantly higher viral load in NPS compared with in oropharyngeal swabs [24], but data on saliva viral load was lacking in this study. Nevertheless, obtaining NPS can cause significant discomfort, especially in young children. There is also a risk of generating aerosol, which increases the infection risk in healthcare workers [25]. Therefore, using saliva for the detection of SARS-CoV-2 virus in children could be considered in the future.

Symptomatic patients had significantly higher NPS and saliva viral loads on admission compared to those of asymptomatic patients. Symptomatic patients who were discharged before 6 July 2020 had longer LOS compared to asymptomatic patients. These patients were discharged based on two consecutively negative RT–PCR results from NPS samples, and the asymptomatic group remained hospitalized for a mean duration of 16.3 days. Study has shown that asymptomatic patients had lower infectivity than symptomatic patients [26], and data from our study also demonstrated that both NPS and saliva viral load decreased with time. In addition, patients may not be infectious for the entire course of virus detection, as it may not represent infectious viable virus. Future studies should explore the infectivity of asymptomatic children during the later phase of infection by viral culture in order to avoid prolonged hospitalization unnecessarily.

Recent studies reported that adults with severe clinical symptoms had significantly higher viral load in NPS and longer viral shedding period than adults with milder disease [27]. A study also reported that NPS viral load was a predictor for mortality in adults [28]. These studies, however, did not address the association between viral load and clinical severity with respect to immunological profiles, especially in the pediatric population. We observed that patients with total, CD3, and CD4 lymphopenia were more likely to have higher saliva viral load on admission, but not NPS viral load on admission, when comparing with those without lymphopenia. Saliva viral load was inversely correlated with the total, CD3, CD4 and B lymphocyte counts on admission. An earlier study showed that angiotensin-converting enzyme (ACE) 2-positive epithelial cells lining salivary gland ducts were early target cells of SARS-CoV [29]. The salivary gland is an important immune organ that is constantly exposed to pathogens. It contains antigen-presenting dendritic cells, and is a site of B cell activation, isotype switching and the induction of antigen-specific cytotoxic T lymphocytes [30]. Zhou et al. reported that during the acute phase of SARS-CoV-2 infection in adults, despite neutralizing antibodies rapidly and abundantly being generated, there was impaired dendritic cell and T cell immune responses and delayed RBD- and NP-specific T-cell development [9]. Wang et al. demonstrated that the total lymphocytes, CD4+ and CD8+ T cells, B cells, and NK cells were all decreased in adult COVID-19 patients, which was more prominent in severe patients than in those with mild disease [8]. Zhang et al. also reported that adult patients with T cell lymphopenia had worse clinical outcomes [31]. These clinical findings support the interaction between COVID-19 infection, especially via the salivary glands, and the evasion of the immune system.

Findings in this study need to be interpreted with the following caveats. Not all patients in our cohort received all the assessment on admission. Only 22 patients had both saliva viral load and lymphocyte subset measured, and only 33 patients had both NPS viral load and lymphocyte subset measured. Nevertheless, saliva viral load remained significantly associated with longer LOS after adjusting for treatment. In the subgroup analysis, saliva viral load remained to be significantly correlated with lymphocyte subset and performed better than NPS viral load. None of the patients in our cohort developed severe disease, therefore comparison between mild and severe COVID-19 was not possible. Multiple pediatric COVID-19 studies have reported that the majority of children with COVID-19 were either asymptomatic or suffered only mild symptoms. Saliva viral load was not measured in children younger than three years old, as well as in some pre-school children, because they were unable to provide saliva specimen. Future studies should explore whether using commercially available saliva collection devices for infants are suitable for the detection of SARS-CoV-2.

## Conclusion

Saliva viral load appeared to correlate better with clinical symptoms, hospital length of stay and immunological profiles than NPS viral load. Symptomatic children had higher NPS and saliva viral loads on admission, which predicted longer hospital stay regardless of the antiviral treatment. Saliva viral load correlated well with NPS viral load. Patients with total, CD3, and CD4 lymphopenia were more likely to have higher saliva viral load, but not NPS viral load. Both B lymphocyte counts and IgG levels were predictors of hospital LOS. Larger-scale studies are needed in the future to compare the sensitivity and specificity of using NPS versus saliva viral load for the detection of SARS-CoV-2 in children.

## Authors’ Contributions

GT Chua, JSC Wong, KKW To drafted the manuscript. JSC Wong, ISC Lam, JS Rosa Duque, PPK Ho, FYS Yau, WH Chan, YW Kwan recruited the patients and collected the specimen. WHS Wong performed the statistical analysis. Cyril CY Yip and Anthony CK Ng performed the virological laboratory analysis. Kate FS Leung, Joyce HY Kwong, PT Wan and Kelly Lam performed the haematological and immunological analysis. Janette Kwok, Marco HK Ho, Godfrey CF Chan, Yu Lung Lau, Patrick Ip, and Mike YW Kwan critically appraised the manuscript as well as supervised the study. All authors agreed on and finalized the current manuscript.

## Role of Funding Source

This study was partly supported by the Consultancy Service for Enhancing Laboratory Surveillance of Emerging Infectious Diseases and Research Capability on Antimicrobial Resistance of the Department of Health of the Hong Kong Special Administrative Region; the donations of Richard Yu and Carol Yu, May Tam Mak Mei Yin, the Shaw Foundation Hong Kong, Michael Seak-Kan Tong; and the Collaborative Research Fund 2020/21 and One-off CRF Coronavirus and Novel Infectious Diseases Research Exercises.

## Ethics Committee Approval

This study was approved by the University of Hong Kong/Hospital Authority Hong Kong West Cluster Institutional Review Board (reference number: UW 20-292), the Kowloon West Cluster Research Ethics Committee (reference number: KW/FR-20-086) and the Kowloon Central Cluster Research Ethics Committee (reference number: KC/KE-20-0140/ER-1).

## Supplementary Material

Supplementary_File.docx
