# Supplementary material for: Saliva viral load better correlates with clinical and immunological profiles in children with coronavirus disease 2019
Source: Emerg Microbes Infect. 2021 Feb 20;10(1):235–41. doi: 10.1080/22221751.2021.1878937 (PMC7899683; doi:10.1080/22221751.2021.1878937)
Supplement: Supplementary_File.docx [file TEMI_A_1878937_SM2035.docx]

**Supplementary File**

**Table 1. Comparison of mean nasopharyngeal swab (NPS) viral load and saliva viral load with clinical symptoms.**

|  |  | Mean NPS viral load (IQR) | | p-value | Mean Saliva viral load (IQR) | | | p-value |
| --- | --- | --- | --- | --- | --- | --- | --- | --- |
| Fever | N | 6.2 (4.3-7.8) | | 0.083 | 5.1 (3.9-5.9) | | | 0.110 |
|  | Y | 6.9 (5.7-8.3) | |  | 5.9 (4.9-6.5) | | |  |
| Cough | N | 6.5 (4.8-8.1) | | 0.818 | 4.9 (3.9-5.8) | | | **0.002** |
|  | Y | 6.6 (5.7-8.1) | |  | 6.4 (5.1-7.0) | | |  |
| Sputum | N | 6.4 (4.9-8.1) | | 0.623 | 5.2 (3.9-6.1) | | | **0.036** |
|  | Y | 6.8 (5.7-8.2) | |  | 6.4 (5.0-8.7) | | |  |
| Running nose | N | 6.6 (5.2-8.1) | | 0.233 | 5.1 (3.9-6.1) | | | 0.055 |
|  | Y | 6.0 (4.4-7.9) | |  | 6.2 (5.0-7.1) | | |  |
| Sneeze | N | 6.5 (5.0-8.1) | | 0.452 | 5.3 (4.2-6.2) | | | - |
|  | Y | 5.5 (4.7- ) | |  | - | | |  |
| Stuff nose | N | 6.5 (5.0-7.9) | | 0.486 | 5.3 (4.2-6.2) | | | - |
|  | Y | 7.2 (4.7- ) | |  | - | | |  |
| Ageusia | N | 6.5 (5.1-8.1) | | 0.257 | 5.3 (3.9-6.1) | | | 0.322 |
|  | Y | 5.5 (4.3-6.8) | |  | 6.2 (4.7- ) | | |  |
| Anosmia | N | 6.5 (4.9-8.1) | | 0.994 | 5.3 (3.9-6.1) | | | 0.481 |
|  | Y | 6.5 (6.0- ) | |  | 6.0 (4.7- ) | | |  |
| Inappetence | N | 6.5 (5.0-8.1) | | 0.611 | 5.4 (4.0-6.2) | | | 0.797 |
|  | Y | 6.2 (4.6-8.3) | |  | 5.1 (4.8- ) | | |  |
| Headache | N | 6.4 (4.9-8.0) | | 0.493 | 5.2 (3.9-6.1) | | | **0.008** |
|  | Y | 7.0 (5.6-8.2) | |  | 7.3 (5.2-9.4) | | |  |
| Dizziness | N | 6.5 (4.9-8.1) | | 0.745 | 5.4 (4.4-6.2) | | | 0.444 |
|  | Y | 6.1 (5.7- ) | |  | 4.5 (3.6- ) | | |  |
| Vomit | N | 6.5 (4.9-8.1) | | 0.946 | 5.3 (4.1-6.1) | | | 0.387 |
|  | Y | 6.5 (4.8-8.0) | |  | 6.7 ( ) | | |  |
| Diarrhea | N | 6.5 (5.0-8.1) | 0.608 | | | 5.3 (4.0-6.1) | 0.897 | |
|  | Y | 6.1 (4.6-7.5) |  | | | 5.5 (4.8- ) |  | |
| Stomachache | N | 6.5 (4.9-8.1) | 0.647 | | | 5.3 (4.2-6.2) | - | |
|  | Y | 7.1 (6.7- ) |  | | | - |  | |
| Chest pain | N | 6.5 (4.9-8.1) | 0.917 | | | 5.3 (3.9-6.1) | 0.076 | |
|  | Y | 6.6 (6.6- ) |  | | | 6.9 (5.4- ) |  | |
| Tiredness | N | 6.5 (5.1-8.1) | 0.478 | | | 5.3 (3.9-6.2) | 0.718 | |
|  | Y | 6.1 (3.8-8.1) |  | | | 5.6 (4.7-5.3) |  | |
| Myalgia | N | 6.5 (4.9-8.0) | 0.315 | | | 5.3 (4.2-6.2) | - | |
|  | Y | 8.3 ( ) |  | | | - |  | |

**Table 2. Correlation analysis between NPS viral load and immunological profiles**

|  | r | p value |
| --- | --- | --- |
| Total Lymphocytes | -0.036 | 0.759 |
| Total T cells | -0.187 | 0.298 |
| CD4 T Cells | -0.196 | 0.274 |
| CD8 T Cells | -0.158 | 0.379 |
| B cells | -0.231 | 0.196 |
| NK cells | 0.032 | 0.858 |
| Immunoglobulin G | -0.224 | 0.088 |
| Immunoglobulin A | -0.191 | 0.147 |
| Immunoglobulin M | -0.068 | 0.609 |

**Table 3. Correlation analysis between saliva viral load and immunologicla profiles**

|  | r | p value |
| --- | --- | --- |
| Total Lymphocytes | -0.430 | **0.001** |
| Total T cells | -0.553 | **0.005** |
| CD4 T Cells | -0.603 | **0.002** |
| CD8 T Cells | -0.406 | **0.049** |
| B cells | -0.482 | **0.017** |
| NK cells | -0.416 | **0.043** |
| Immunoglobulin G | -0.236 | 0.127 |
| Immunoglobulin A | 0.062 | 0.692 |
| Immunoglobulin M | 0.032 | 0.843 |

Supplementary Figure 1. Correlation analysis between log_10_ nasopharyngeal swab, log_10_ saliva viral load and lymphocyte subsets.

Correlation analysis for (a) Log_10_ nasopharyngeal swab (NPS) viral load with total lymphocyte count  (r=-0.36, p=0.759); (b) Log_10_ saliva viral load with total lymphocyte count [r=-0.43, p=0.001]; (c) Log_10_ NPS viral load with T cell Count  (r=-0.187, p=0.298); (d) Log_10_ saliva viral load with T lymphocyte count [r=-0.55, p=0.005]; (e) Log_10_ NPS viral load with CD4 count  (r=-0.196, p=0.274) (f) Log_10_ saliva viral load with CD4 lymphocyte count (r=-0.6, p=0.002); (g) Log_10_ NPS viral load with CD8 lymphocyte count  (r=-0.158, p=0.379) (h) Log_10_ saliva viral load with CD8 lymphocyte count (r=-0.41, p=0.049) (i) Log_10_ NPS viral load with B lymphocyte count (r=-0.231, p=0.196); (j) Log_10_ saliva viral load with B lymphocyte count (r=-0.482, p=0.017); (k) Log_10_ NPS viral load with NK lymphocyte count (r=0.032, p=0.858) (l) Log_10_ saliva viral load with NK lymphocyte count (r=-0.416, p=0.043)
